# Photocatalytic Activity of Titanium Dioxide Nanotubes Following Long-Term Aging

**DOI:** 10.3390/nano11112823

**Published:** 2021-10-24

**Authors:** Stephen Abela, Clayton Farrugia, Ryan Xuereb, Frederick Lia, Edwin Zammit, Alex Rizzo, Paul Refalo, Maurice Grech

**Affiliations:** 1 Faculty of Engineering, University of Malta, MSD 2080 Msida, Malta; clayton.farrugia@um.edu.mt (C.F.); paul.refalo@um.edu.mt (P.R.); Maurice.Grech@um.edu.mt (M.G.); 2 Econetique Ltd., Xewkija Industrial Estate, Xewkija, XWK 3000 Gozo, Malta; research@econetique.com; 3 Institute of Applied Sciences, Malta College for Science Arts and Technology, Triq Kordin, PLA 9032 Paola, Malta; Frederick.Lia@mcast.edu.mt (F.L.); Edwin.Zammit@mcast.edu.mt (E.Z.); Alex.Rizzo@mcast.edu.mt (A.R.)

**Keywords:** photocatalytic surface, long-term aging, reactivation

## Abstract

Anodic titanium dioxide (TiO_2_) nanotubes were found to be active photocatalysts. These photocatalysts possess a high surface area, even when supported, rendering them potential candidates for water treatment. In this work, photocatalytic surfaces were produced by anodizing commercially pure Ti plates using two different electrolyte compositions and correspondingly diverse process parameters. Changes in the physical and chemical stability as well as photocatalytic activity were studied over a fifty-two-week aging process. During this period, the nanotubular surfaces were exposed to flowing synthetic greywater, solar irradiation, and the natural environment. The physical and phase stability of the materials anodized using the organic electrolyte were found to be outstanding and no degradation or change in crystalline structure was observed. On the other hand, materials anodized in the aqueous electrolyte proved to suffer from light-induced phase transition from anatase to rutile. Surfaces synthesized in the organic electrolyte were more resistant to fouling and showed a better tendency to recover photocatalytic activity upon cleaning. In conclusion, the nanotubes produced in the organic electrolyte proved to be stable, rendering them potentially suitable for real-life applications.

## 1. Introduction

Water scarcity affects at least 11% of the European population and 17% of EU territory. This has cost an estimated €100 billion over the past 30 years [1]. Furthermore, changes in weather pattern are becoming more extreme. In 2007, the EU established the Action on Water Scarcity and Drought and identified seven policy options for tackling the issue of water scarcity. Some of these policies involve the following: the removal or restriction of subsidies on water tariffs, thus ensuring a more efficient water use, consideration of additional water supply infrastructures, and the development of water efficient technologies and practices. In spite of this, to date, in Europe an estimated 30% of town water supply is used for toilet flushing [2]. This means that potable water is used where water of a lower quality and including treated greywater would suffice. Treatment of greywater could be carried out in situ using photocatalytic degradation of the pollutants in the effluent from the same residence. This technology allows for the effective treatment of greywater without the addition of chemicals. In fact, such technology has already been shown to be effective [3,4].

Ever since Honda and Fujishima successfully used a TiO_2_ electrode to split water by photoelectrolysis [5], TiO_2_ has become the subject of numerous investigations. Studies are focused on nanoparticles of TiO_2_ as these have properties that are vastly different to those of the bulk and when exposed to UV light in an environment which contains oxygen, they sustain photooxidative and photoreductive reactions that can degrade pollutants in humid air and water [6]. This relatively inexpensive oxide has already found widespread use in self-cleaning glass. Furthermore, TiO_2_ nanoparticles are non-toxic, have a high chemical stability, and are resistant to corrosion, rendering them suitable candidates for water treatment [7]. Moreover, owing to their intrinsic wide band-gap (≈3.0–3.2 eV), these semiconductors can make use of UVA radiation from the solar spectrum to degrade pollutants photocatalytically [8]. This has led to TiO_2_ crystalline nano-powders being used in slurry type photoreactors intended for water treatment [9]. Owing to the small size of the nano-powders, their complete recovery following water treatment tends to be problematic. TiO_2_ powders have been reported to cause adverse effects such as oxidative stress in human cells [10,11] and genetic instabilities in mice [12]. Thus, the recovery and responsible disposal of the nanoparticles has become a priority [13].

Supported catalysts may on the other hand eliminate the potential hazards posed by the titania nano-powders. Anodizing titanium metal is a particularly simple production route for the synthesis of TiO_2_ nanotube layers that are attached to the passive substrate. Furthermore, the anodic nanotubes produced in this way exhibit higher activities compared to commercially available TiO_2_ powders of comparable dimensions [14,15].

Anodic nanotube arrays have been first synthesized by Zwilling et al. back in 1999 [16]. The facile synthetic method is especially attractive since it produces free standing, high aspect ratio ordered layers [17] that were thus the subject of numerous investigations. The shape and properties of these nanotubular arrays depend on the electrolyte mixtures used for their synthesis. The first three generations of electrolytes used for this purpose feature the fluoride ions as pitting initiators. This, under the influence of an applied potential, gives rise to the progressive evolution from pit to nanotube [18,19]. The fluoride ions promote the production of nanotubes with large diameters and tubes of several hundred microns in length [20]. The fourth generation of electrolytes replaced fluoride ions with chloride ions [21]. The resulting tube morphologies are usually irregular and likely have a lower surface area than those produced using baths containing fluoride salts [22,23]. Nanotubes with similar morphologies can also be produced using sol-gel and hydrothermal deposition methods. However, these coatings are known to gradually leach TiO_2_ into the water being treated and have a lower photoelectrochemical activity when compared to anodized surfaces [24].

The degradation of biological and chemical contaminants using nanotubular anodized titania under artificial UVA irradiation has been amply reported in literature [25,26,27]. The effect of sunlight as a UV source has also been investigated but to date the potential of these materials for water treatment remains unexploited [28]. A small-scale solar reactor was produced by Gomes. In this study sunlight was found to degrade parabens more effectively than UVA blacklights [29]. McMichael investigated the photoelectrocatalytic disinfection ability of anodic TiO_2_ nanotubes under solar irradiation [30] whilst Kim et al. studied the efficiency of these arrays in breaking down different organic compounds [31].

In this work, we assessed the long-term performance of different nanotubular arrays produced using selected electrolytes and synthesis methods reported in literature. The nanotubular arrays were subjected to a continuous flow of synthetic greywater while exposed to ambient conditions, in an experiment carried out over a 52-week period. The ability of the aged nanotubular arrays radiated with UVA to degrade organic pollutants, was then assessed using a methylene blue solution. The effect of aging duration and deposits that form on the surface, possibly clogging or fouling the surface was thus determined. Changes in the crystal structure and surface morphology were also investigated. This extended experiment is believed to provide a realistic platform for the evaluation of the candidate photocatalytic surfaces, and method of their production, for industrial applications such as the micro greywater treatment reactor prototype being developed by the authors [32].

Methylene blue was used to represent dyes and other organic contaminants like the surfactant Sodium dodecyl sulfate that bleeds out from garments during laundry [33] and is present in most personal care products. The wastewater concentration of surfactants is subject to stringent limits [34] and thus its control is crucial. Given the widespread use of paracetamol, and that irrigation is one of the potential uses of recycled greywater, then investigations into the potential of its removal is of importance [35,36]. The ability of these photocatalytic materials to breakdown bacteria commonly found in greywater was also investigated. Results from these investigations are reported in separate publications by the same authors [37,38].

## 2. Methodology

The stability of the materials when subjected to long term use in photocatalytic greywater treatment was assessed. In order to account for the complexity of the day-to-day variations during true would-be deployment, the test was conducted in a scaled down replica of the reactor under development [32]. The test was conducted under solar radiation for a period of 52 weeks. The test setup and the characterization exercises conducted are discussed in this section. The methods by which the photocatalytic activity of both unaged and aged materials is assessed are reported. The design and the rationale behind the aging exercise are described in detail.

### 2.1. Material Synthesis

Two electrolytes were chosen for the “etching” of the nanotube varieties considered in this study. These were 1 M sodium sulfate [39,40] and ethylene glycol, both containing fluoride ions [41,42]. These will hereafter be referred to as TiO_2_NT-S and TiO_2_NT-O respectively. The nanotube arrays were produced by anodizing commercially pure (99.6%) titanium (Grandis, Rancho Santa Margarita, CA, USA) using a two-electrode setup. Just before anodizing, the plates were sonicated in acetone for 10 min rinsed in isopropanol and sonication again in deionized (DI) water. The two equally sized titanium plate electrodes (26 × 26 × 1 mm^3^) were immersed in the electrolyte and held 30 mm apart. Anodizing was conducted under potentiostatic conditions using a Delta Elektronika SM66-AR-110 power supply. During anodizing the electrolyte was kept in constant circulation.

The aqueous bath consisted of a solution of 0.5 wt% sodium fluoride (Sigma Aldich, Taufkirchen, Germany) in 1 M sodium sulfate (Sigma Aldrich). In this case, the anodizing process was rather sluggish and required 6 h of processing to produce a nanotube layer thickness of just 1.5 μm. The maximum anodizing potential that could be applied was just 20 V. All attempts to apply a higher potential to accelerate the process invariably resulted in the loss of the nanostructure.

The organic bath used consisted of 3 wt% deionized (DI) water, 0.5 wt% ammonium fluoride (Sigma Aldrich, Taufkirchen, Germany) and ethylene glycol (Scharlab, Barcelona, Spain) as balance. In this case, a potential of 70 V could be safely applied, provided the bath was suitably cooled. The process was conducted for just 1 h, yielding far longer nanotubes than those obtained with the aqueous bath.

With both baths, the final step of the anodizing process was a gentle rinse with acetone in order to remove the electrolyte from the surface of the samples. These arrays were subsequently annealed at 450 °C for 2 h. The annealed samples were finally sonicated for 5 min in isopropanol and air dried. A set of TiO_2_NT-O samples were decorated with silver nanoparticles. This was achieved by heating 200 mL of 5 mM trisodium citrate under reflux in a three-neck flask until the solution was boiling off. A total of 1 mL of 0.25 mM silver nitrate solution was added and the solution was left boiling for a further 20 min. The solution was left to cool to room temperature and diluted by half using Milli-Q water. The nanotube samples were dipped in the solution and left for 24 h. The samples were then rinsed gently with deionized water [40,41]. These samples are referred to as TiO_2_NT-Ag.

### 2.2. Aging Test Setup

This experiment follows a lab-based experiment in which greywater flowed on the surface of the samples in an open channel configuration. The problem with such a test setup was that in an open channel water could flow freely and at the boundary layer the fluid velocity is zero. This configuration was not representative of water recycling installations units in urban areas in which case the water must be contained in order to minimize cross-contamination and water loss through evaporation. In fact, in a closed channel water flows differently especially if the flow cross section is minimized to ensure a high probability of contact with the active surface. In this case water forms a film, sandwiched between a UVA transparent window and the active surface. Moreover, the constantly changing atmospheric condition can affect the performance of the active surface. It was therefore deemed necessary to compliment the lab-based experiments with a long-term aging experiment subject to environmental conditions. For this purpose, an experimental reactor was designed to contain the test coupons.

**Figure 1:**
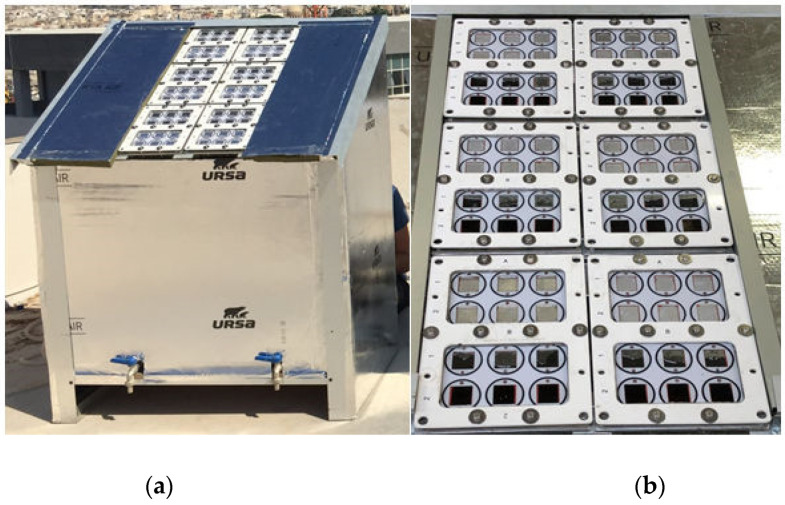
(**a**) Aging unit and (**b**) tile arrangement.

**Figure 2:**
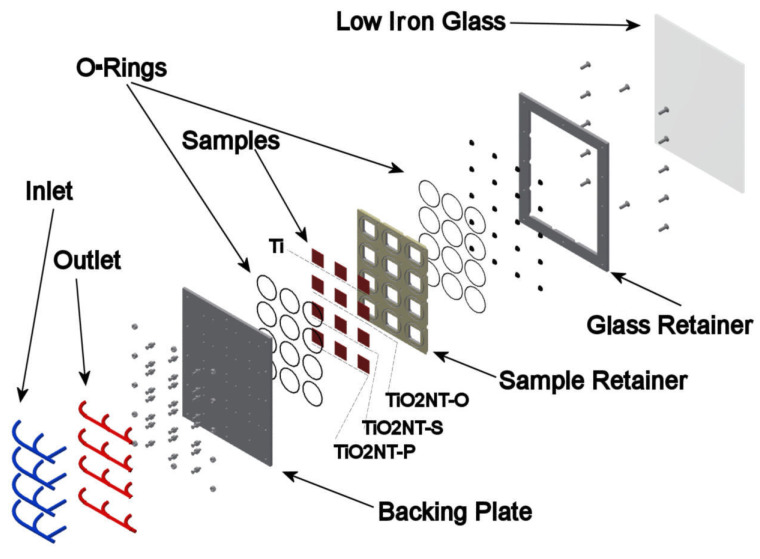
Exploded view of sample cell tile.

The experimental setup shown in Figure 1 was designed such that each coupon experiences similar greywater flow conditions as those calculated for the micro water treatment prototype under development [42]. Each coupon has its own inlet and outlet so that the flow in each cell is independent of that in any of the other test coupon. Each sample envelope was created by two O-rings sitting on the sample retainer plate, which is sandwiched between the sample backing plate and the glass to create a 30 × 30 × 2 mm^3^ chamber for each sample, as shown in Figure 2. The dimensions of the samples used were 26 × 26 × 1 mm^3^. The thickness of the water film above the active surface was limited to a maximum of 1 mm. The aging unit was filled from an inlet located at the bottom, thus ensuring that any air in the unit was displaced and the unit completely flooded each chamber. The aging setup included four twenty-five litre tanks, one for each for the three materials under investigation and Ti control plates. Each tank contained a circulation pump which circulated the greywater over the materials being tested. To avoid debris from dispersing the incident sunlight and preventing it from reaching the photocatalyst surface, a 5 µm and a 2 µm cartridge filters were used. Filtering was also necessary as accumulation of debris could also result in blockage leading to changes in pressure within the system, and potentially lead to glass breakage. A flow rate of 146 L/h was maintained through each of the four circuits for the whole duration of the experiment. During mounting, samples were removed from their protective storage bag, using latex or nitrile gloves, cleaned by immersion in isopropyl alcohol (IPA) or ethanol, dried and placed in their specific pocket within the tile.

The aging unit consists of six tiles, one for each aging interval. Each tile contains the whole complement of coupons for a specific period and three samples of each material being tested and three blank Ti plates. The arrangement is presented in Figure 1. The aging unit was first tested for leaks for 2 days by flowing tap water over blank coupons. During this period the pumps were throttled so as to achieve the design flow rate. The blank coupons were then removed and the reservoirs thoroughly cleaned and dried. The tiles were dismantled, and any residual water was dried using a clean cloth. Finally, the test coupons and a set of control blanks were mounted in the tiles as indicated in Figure 2.

### 2.3. Preparation of Synthetic Greywater

To ensure greywater homogeneity for the whole duration of the aging period, synthetic greywater was used. The chemical composition of the greywater was based on a modified Thompson [43] recipe. The chemical composition of the solution used is presented in Table 1. The working solution was prepared overnight so as to ensure that at the time of mixing all the salts were in solution. Kaolin and cellulose (both of which would be filtered out in practice) were not added in the final mixture, as these insoluble compounds increase the risk of blocking the cells. The final solution was obtained by diluting the working solution with deionized water and filtering with 0.45 µm cellulose filter paper.

The initial bacterial composition of the greywater used was restricted to only 4 bacterial species, those most commonly found in greywater. In order to prevent any health hazards, the bacteria selected are all of biosafety level 1, namely: *Brevundimonas diminuta* (ATCC^®^ BAA-2474, Washington, DC, USA), *Escherichia coli* (ATCC^®^ 8739™, Washington, DC, USA), *Staphylococcus epidermidis* (ATCC^®^ 12228™, Washington, DC, USA), and *Klebsiella aerogenes* (ATCC^®^ 13048™, Washington, DC, USA). Moreover, 5 × 10^4^/1 mL of each was added to the synthetic greywater after water changes held once a week. It is to be noted that the scope of this experiment was not to analyze the changes in the greywater solution, but to investigate changes to the active surface when exposed to the operating conditions over extended periods of time. Prior to each water change, the setup was drained and filled with a 100 ppm of peroxide solution. This procedure was necessary to stop the small diameter tubes and channels from being blocked with biofilm growth.

During the first four weeks of operation of the aging unit, the water temperature, pH, conductivity, and turbidity were monitored on alternating days using a Hannah HI98194 Multiparameter Instrument and a Hach DR3900 Laboratory Spectrometer. Other parameters were measured three times weekly to understand the changes in the quality of the greywater and establish the water change regime needed so as to maintain the chemistry of the greywater within specified units and establish a safe water replacement interval. The first greywater sample was collected and analyzed after 24 h of operation, the second after 72 h of operation, and the final sample at the end of the week. A 200 mL aliquot was withdrawn from the bottom of the four recycling tanks and filtered through a grade 1 filter paper in order to remove any deposits. These were used to measure calcium and total hardness, nitrate and nitrite, Total Phosphate, COD, Surfactants (Cationic, Anionic, and Neutral), chloride, ammonia, and the TOC. Samples of water that could not be analyzed on the same day, were labelled and stored at −10 °C or treated as per the water testing kit requirements.

### 2.4. Sample Handling and Testing

Each window contained a complete set of samples (three repeats of three different materials and a control) planned to be sampled as a single batch at a predetermined time. Sampling extraction occurred after aging periods indicated in Table 2 and coincided with one of the test rig water maintenance periods. Sample extraction did not affect the samples held in other tiles destined to be opened at a later date. At the end of each test period, the respective tile was disassembled and the coupons stored. The spent tile was cleaned thoroughly with distilled water and loaded with titanium blanks of identical dimensions. This was necessary so as to maintain greywater flow conditions of the experiment the same for all the samples.

#### 2.4.1. Morphological Investigation

Following each extraction, loose debris was carefully removed from the samples and collected in labelled sample bottles. Prior to SEM analysis, the samples were immersed in deionized water, sonicated, and air dried. The surface of the sample was analyzed using a Carl Zeiss Merlin Field Emission SEM equipped with an Ametek EDAX trident system for EDS and microscopic analysis. Any change in morphology and surface degradation was recorded. Finally, the crystal structure of the aged surfaces was investigated using a Rigaku Ultima IV Cu-Source X-ray Diffractometer and a step size of 0.02 s with a dwell time of 1 s. The exposed area was 1 × 1 μm^2^ which was scanned for 100 live seconds. Diffractograms of materials aged for different periods were obtained using scanning angles between 20° and 90°.

#### 2.4.2. Methylene Blue Dye Degradation Tests

When all samples were extracted, they were rinsed with DI water, dried in hot air, and stored in a dry and dark environment for three months to allow for the organic growth to decompose naturally. Following the storage, coupons were prepared for the methylene blue dye degradation tests by immersion in deionized water and irradiated with a UV source with a nominal wavelength between 350 and 400 nm for a period of 3 h and an irradiance of 1 mW/cm^2^. This procedure removed any hydrocarbons present on the sample surface [44]. The procedure adopted to quantify the photocatalytic activity was as described in ISO 10678:2010 [45]. The samples were transferred to a borosilicate beaker containing 10 mL of 1.5 × 10^−5^ M of methylene blue (MB) solution. The samples were kept in the dark, allowing for the adsorption/desorption equilibrium between sample and solution to be reached. The absorbance of the solution was read every 15 min using a UV–VIS spectrophotometer. This procedure was repeated until a stable absorbance value was obtained. The sampled aliquot was returned to the beaker and kept in the dark for a further 30 min. This was then transferred to an Opsytec BS-02 chamber with a UV MAT controller. The absorbance of the solution after irradiation was measured at 30 min intervals over a period of 4 h. A scan range between 500 and 800 nm was used to detect the absorbance using a Shimadzu SolidSpec 3700i.

## 3. Results and Discussion

In this section, the results obtained from tests conducted on the aged samples are described and discussed.

### 3.1. Morphology and Chemical Composition

**Figure 3:**
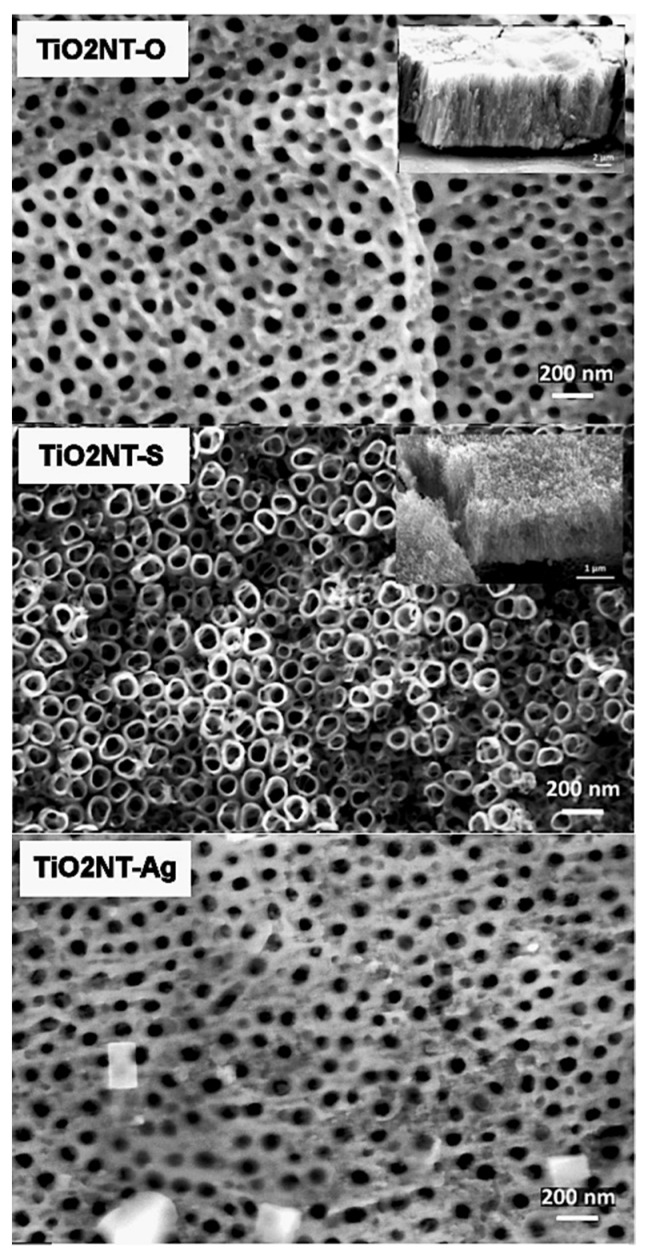
Surface morphology of the unaged samples.

The morphologies obtained using the anodizing methods described above are presented in Figure 3. Table 3 summarizes the main morphological features of the different nanotube arrays. The details for the TiO_2_NT-Ag are not included because these are just modified TiO_2_NT-O samples and share the same characteristics short of the Ag nanoparticle addition. Freestanding nanotubes were formed in all cases except in the case of the TiO_2_NT-O. In the latter case the tubes were superficially joined by TiO_2_ and titanium hydroxide (TiOH_x_) [46]. Given the short processing time, this thin oxide film was not completely etched off but could be removed by sonicating the samples for longer. On the other hand, the surface of the TiO_2_NT-S showed clearly defined tubes with open spaces in between. EDS measurements established the identity of the nanometric Ag particles deposited on the surface of TiO_2_NT-Ag.

**Figure 4:**
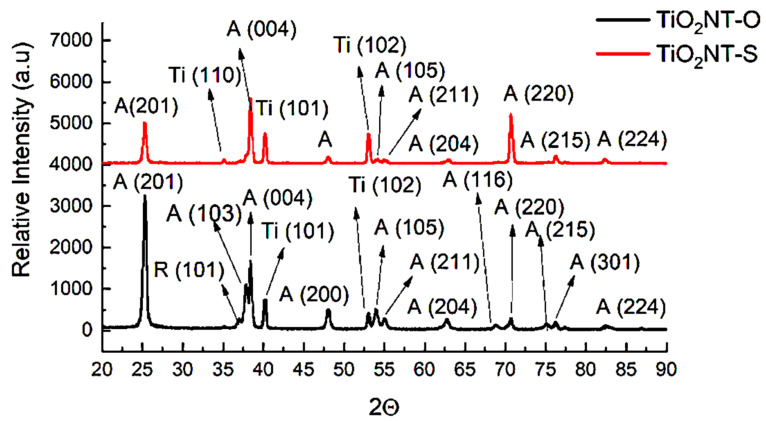
X-ray diffraction patters taken from the anodized surfaces. A = anatase, R = Rutile, Ti = Titanium.

The two anodising processes resulted in the crystalline structures presented in Figure 4. This plot shows main peaks of anatase and titanium indicating that these are the major constituents of the two materials synthesized. A low intensity rutile shoulder at 37° and another shoulder at 77° were recorded for TiO_2_NT-O, indicating that some rutile is still present in this structure despite the material being annealed at 450 °C to consolidate its structure. The presence of this polymorphic structure is believed to be beneficial as it has been reported to have a higher visible light absorbance than the anatase structure. On the other hand, anatase is more active in the UV region of the light spectrum. Thus, a heterostructure composed of the two, could have a higher solar photocatalytic activity than the separate phases on their own as the former can make use of a wider portion of the solar spectrum [47]. At the same time, the presence of rutile could be detrimental to the performance of this material as it is also known to promote the recombination of charged carriers [48].

The peak at 25° corresponding to the TiO_2_NT-S plot is less intense that the counterpart pertaining to the TiO_2_NT-O. This contains peaks from different planes of anatase, namely, (103) and (004) which are manifested in a bifurcated peak at around 38°. The higher substrate detection in this case is attributed to the thinner anodized film resulting from the sluggish reaction when using the water electrolyte. Such electrolyte is also believed to give rise to a specific growth in the (220) direction. When the organic electrolyte was used, the prevalent growth was in the (201) direction.

**Figure 5:**
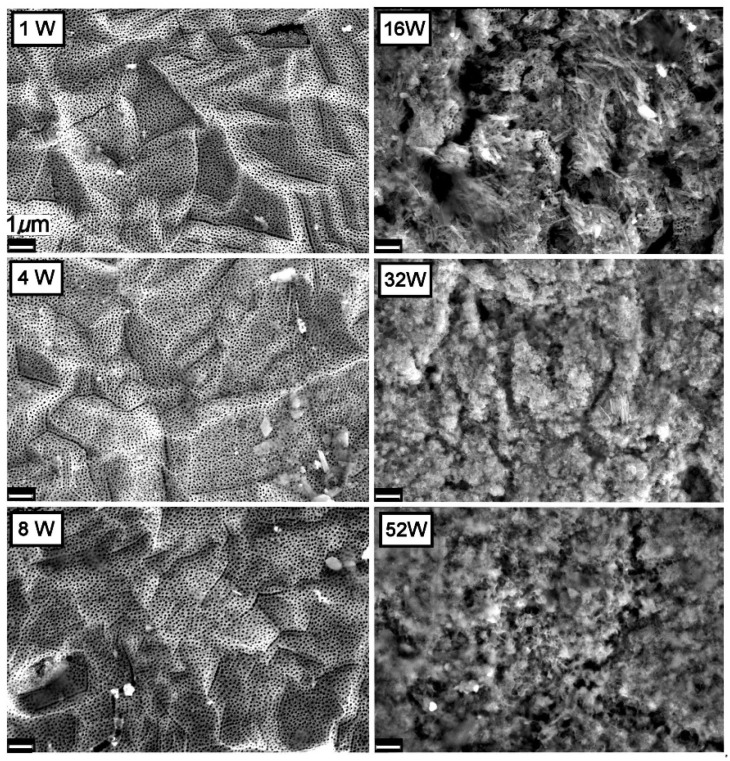
Changes observed on the surface of the TiO_2_NT-O samples when exposed to synthetic greywater under atmospheric conditions.

**Figure 6:**
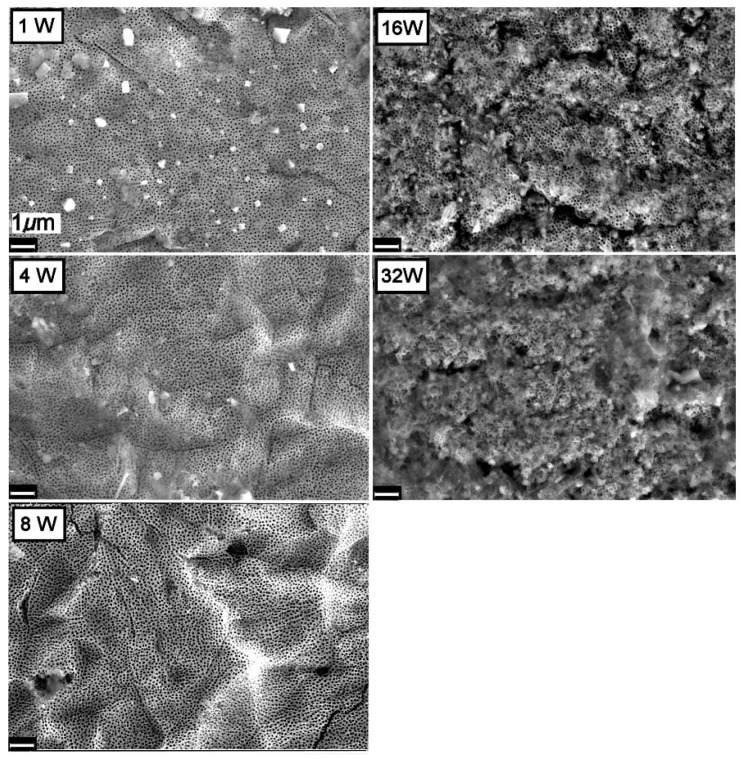
Changes observed on the surface of the TiO_2_NT-Ag sample when exposed to synthetic greywater under atmospheric conditions.

The changes taking place on the surface of the TiO_2_NT-O and TiO_2_NT-Ag samples having synthetic greywater flowing on top while being exposed to atmospheric conditions for periods of up to fifty-two weeks are shown in Figure 5 and Figure 6, respectively. Micrographs for the first three time periods, show that samples of both the TiO_2_NT-O and TiO_2_NT-Ag are mostly free from fouling with the TiO_2_NT-Ag picking up some deposits at four weeks which is no longer visible following eight weeks exposure. The number of silver nanoparticles on this sample surface also decrease with time and are almost completely absent following eight weeks of aging. After 16 weeks of aging both surfaces exhibit a drastic change in surface morphology and extensive areas of the samples are covered with growth (opportunistic organisms from the atmosphere, possibly algae). ImageJ analysis of the SEM images showed that in the case of the TiO_2_NT-O samples, circa 95% of the surface is covered with growth. In comparison the TiO_2_NT-Ag surface fares a bit better and only 74% of the surface was covered. After 32 weeks of aging both the TiO_2_NT-O and TiO_2_NT-Ag samples are completely covered. The slightly better resistance to fouling of theTiO_2_NT-Ag is attributed to the presence of Ag^+^ ions on the surface which are known to have antimicrobial properties [49].

**Figure 7:**
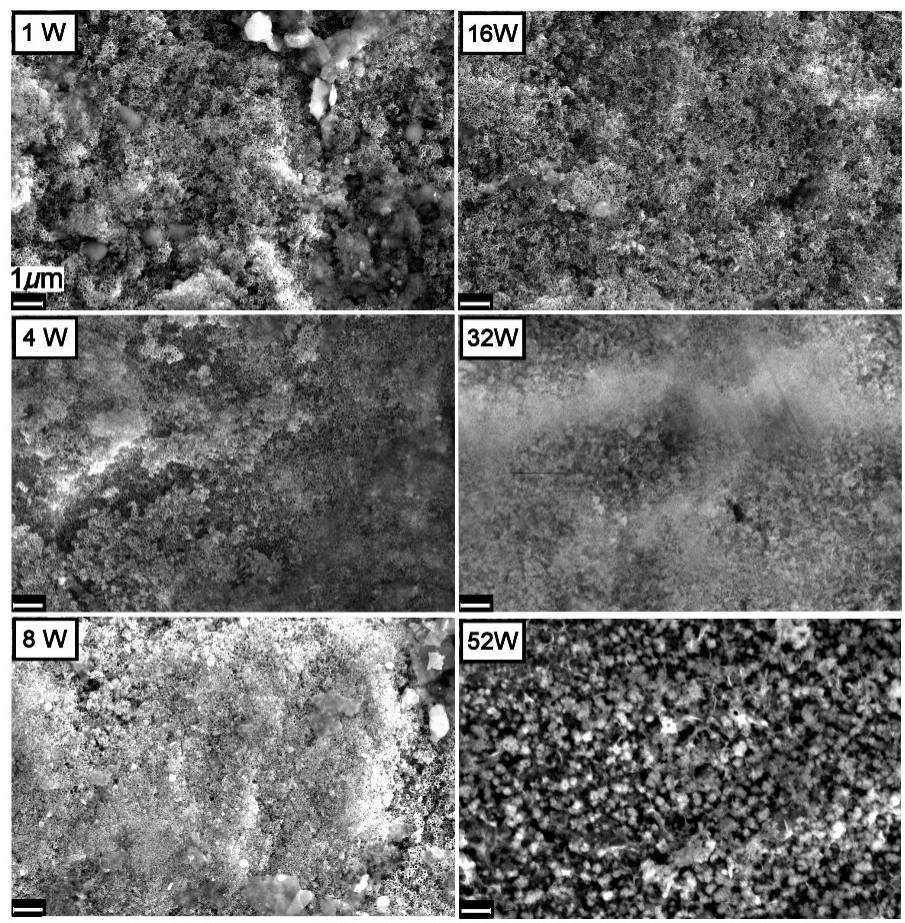
Changes observed on the surface of the TiO_2_NT-S sample when exposed to synthetic greywater under atmospheric conditions.

In contrast to the two other materials under investigation, the TiO_2_NT-S samples were completely covered with debris after just one week of exposure, Figure 7. Those nanotubes sticking out of the growth were also heavily soiled. SEM images of samples aged for 4, 8, and 16 weeks show no visible change in surface morphology. With further exposure however, there is a substantial increase in growth resulting in a thick layer that completely envelopes the surface. The stark difference in behavior between the TiO_2_NT-S and the other two materials under investigation is believed to stem from the original nanotube tip morphology. From Figure 3, it is clear that the nanotube tips of the TiO_2_NT-S samples are very clearly defined. Standalone tubes with a thin wall protruding from the surface at different heights give rise to a very rough surface. In contrast, the samples anodized in the organic electrolyte (TiO_2_NT-O and TiO_2_NT-Ag) present a very flat pitted surface with the tube tips “glued together” by TiO_2_ and titanium hydroxide (TiOHx) residue. It is believed that the presence of this residue on the surface considerably reduces the opportunity for microorganisms of getting a foothold on the surface, but does not significantly interfere with the photocatalytic activity of the surface.

**Figure 8:**
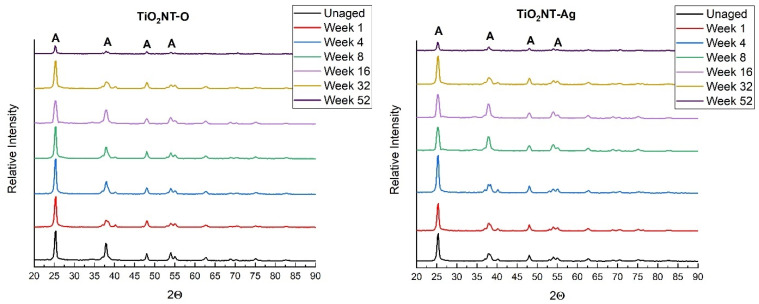
XRD diffraction graphs taken on the samples anodized in the organic electrolyte and aged for different durations.

**Figure 9:**
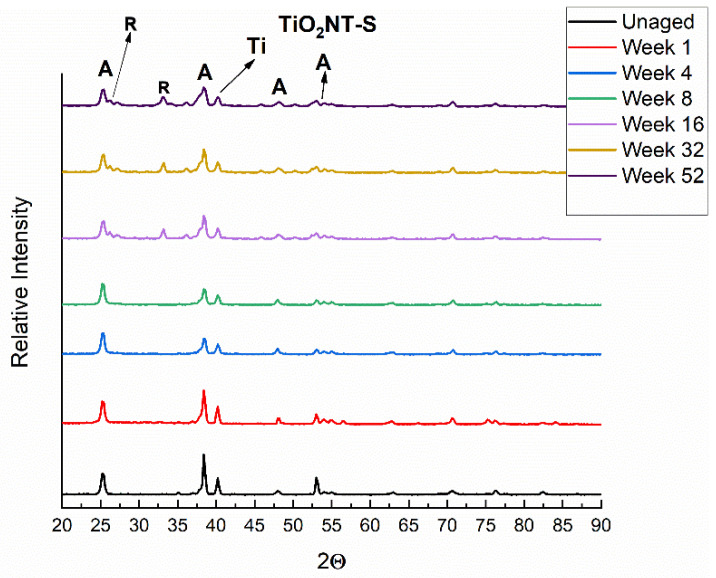
XRD diffraction plots of samples anodized in the aqueous electrolyte and aged for different time periods.

The effect of aging on the crystal structure of the samples was investigated by conducting a series of XRD scans on samples aged at the preselected timepoints. Data from samples anodized in the organic electrolyte are presented in Figure 8. These results show that the position and relative intensities of the peaks remain unchanged (within the limits of experimental errors), showing that there are no detectable changes in the crystal structure of the anodized surface during aging. The XRD results obtained for the samples anodized in the aqueous solution, presented in Figure 9, show that in this case there is a slight change in structure even after short exposures. This is corroborated by the marked reduction of the relative intensity of the anatase (105) peak at 57°. Furthermore, in the case of samples exposed for sixteen weeks or more, two new (101) rutile peaks were detected at 25.1° and 36°. This change in structure indicates that the anodized surface produced in the aqueous electrolyte is not as stable as that produced in the organic electrolyte.

The light-induced phase transition of TiO_2_ nanoparticles from anatase to rutile structure was reported in literature [50]. According to the authors this transition is likely to take place under oxygen-deficient conditions. The transition mechanism includes oxygen adsorption and desorption phenomena with the involvement of surface oxygen vacancies and F centers. The phase transition is initiated by intragap irradiation (with the exception of the red light) that acts as TiO_2_ surface sensitizer. This promotes the activation of the surface and the nucleation of rutile crystallites.

### 3.2. Effect of Aging on the Photocatalytic Activity

**Figure 10:**
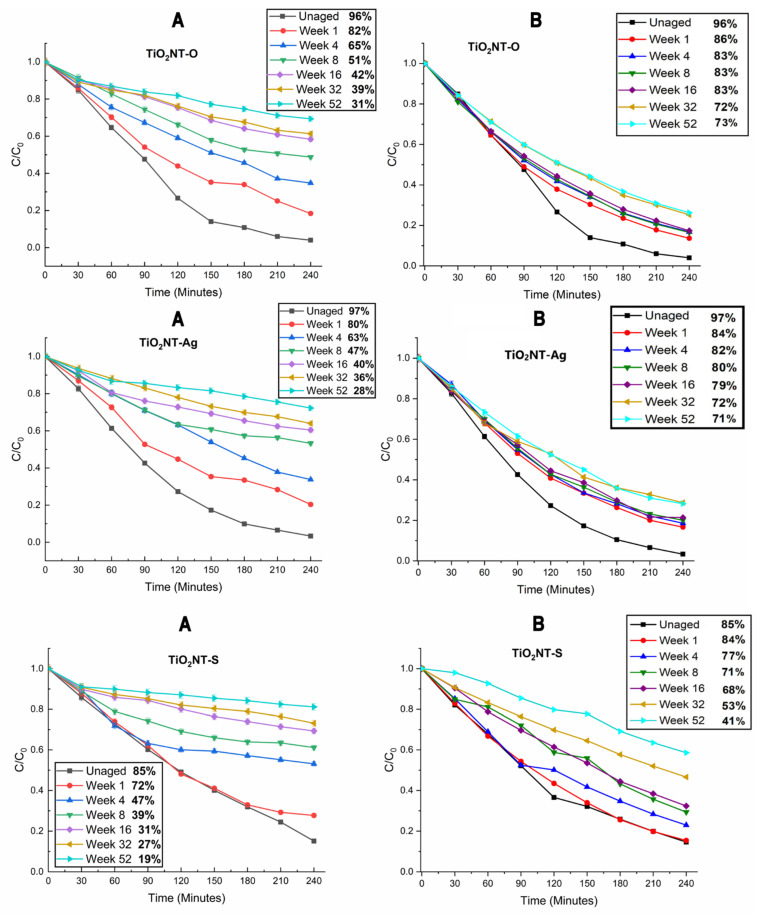
Methylene blue dye reducing tests conducted on the as-collected samples (**A**) and samples dried for 3 months (**B**).

The changes in the photocatalytic activity resulting from different periods of exposure to the environment and synthetic greywater were assessed using a modified methylene blue test, described in Section 2.4.2. Results from this test are illustrated in Figure 10.

The results obtained show that under the aging conditions, the ability of the samples to photocatalytically degrade methylene blue has steadily decreased with aging time. Despite appearing pristine, the TiO_2_NT-O and TiO_2_NT-Ag samples showed a measurable drop in performance even after one week of exposure. On the other hand, after one week, the performance of the TiO_2_NT-S surface remained practically the same despite the surface appearing heavily fouled. With further aging however all samples suffered a massive drop in performance losing half of their effectiveness after just eight weeks of aging and becoming practically ineffective (C/C_o_ of 0.8) by the end of the aging experiment.

**Figure 11:**
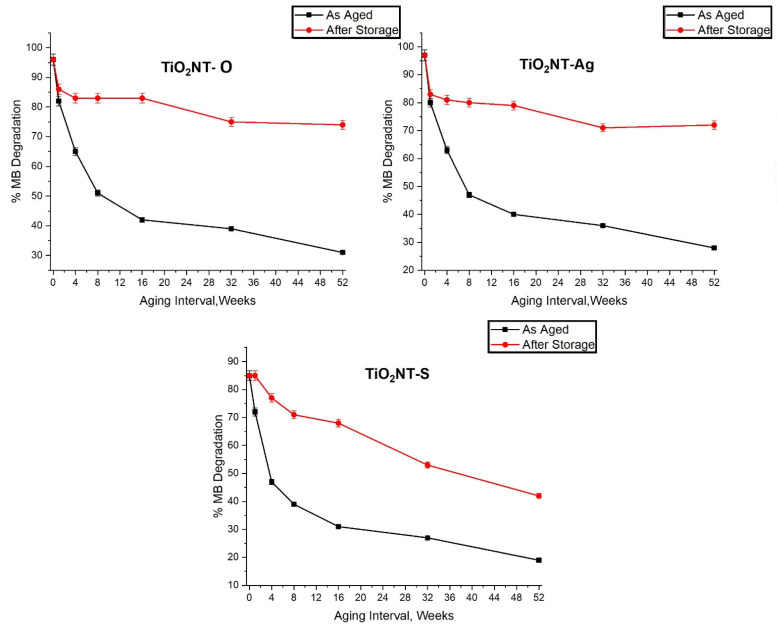
Change in Photocatalytic activity with sample aging duration.

After analyzing the data obtained, it was hypothesized that the loss in performance experienced by samples is mostly caused by the organic build-up on the surface with aging. The TiO_2_NT-O and TiO_2_NT-Ag samples in particular did not show any change in crystal structure. In order to test this hypothesis, the samples needed to be cleaned without damaging the nanostructured surface. In an attempt to achieve this, samples were stored in a dark dry environment for three months so that the organisms on the surface dry and shrink to become easier to dislodge with DI water. The test described in Section 2.4.2. was repeated and the results are shown in Figure 10B. This crude way of cleaning the surface is far from perfect but it was sufficient for the TiO_2_NT-O and TiO_2_NT-Ag samples to regain much of their photocatalytic activity with respect to the degradation of the MB dye. It is interesting to note that even the performance of the most heavily aged samples became close to the lightly soiled samples. In fact, TiO_2_NT-O samples aged for 4–16 weeks samples yielded a C/C_o_ between 0.114–0.17 and those aged for 32–52 weeks, gave C/C_o_ values of 0.25–0.26. Moreover, TiO_2_NT-Ag samples aged for 4–16 weeks had a C/C_o_ value ranging 0.16–0.21 and a C/C_o_ value ranging between 0.28 and 0.29 after 32–52 weeks of aging. In contrast the TiO_2_NT-S samples experienced a more moderate recovery. This is particularly true for samples aged for 16, 32, and 52 weeks which, as discussed in Section 3.1, experience an anatase to rutile transformation. The drop in photocatalytic activity for the three surfaces is shown in Figure 11.

## 4. Conclusions

The vast number of parameters which are impossible to control in real life cannot be accurately simulated in a lab experiment. This is especially true for equipment which makes economic sense only if it can be used for a long period. The diurnal and seasonal variation and the presence of opportunistic organisms can play a significant influence on the functionality and efficiency of the solar photocatalytic treatment unit. From the results obtained in this investigation it can be concluded that in all cases water erosion plays no detectable part. Reducing the number of openings in the water tank is imperative as this limits the ingress rate of opportunistic organisms but unfortunately it also results in a reduced oxygen content in the greywater. This low oxygen content not only reduces the effectiveness of the photocatalytic process, but it also results in the possibility of light-induced phase transition of anatase TiO_2_ to rutile [50], as experienced with the TiO_2_NT-S samples in this study. Samples anodized in the organic solution has proven to be particularly stable and immune to this transformation reaction. Moreover, the residue clogging the spaces in between the tubes not only serves as support but it also helps to slow down the formation of biological growth. Finally, it is clear that these materials do have the potential of use in water recycling technologies, however for this to become an economically viable option, a cheap and ecological way of cleaning the active surface in situ has to be engineered.

The long natural drying process employed in this work proved to be effective in regenerating the surfaces even if it is not a practical process. The experiment did, however, provide valuable information as to how regeneration can be achieved. Hypothetically, artificial drying may regenerate surfaces effectively in a much shorter period of time rendering the process viable. Furthermore, regeneration after short aging durations appears to be highly beneficial.

## Figures and Tables

**Table 1:** Chemical composition of synthetic greywater.

Chemical	Concentration in the Final Solution mg/L	Working Concentration 1:100 Preparation of 10 L Stock (g)
Ammonium Chloride	12.2	12.2
Calcium Carbonate	2.1	2.1
Calcium Chloride	63.5	63.5
CAPB	4.0	4.0
Polyquaterium-10	4.0	4.0
Magnesium Sulfate anhydrous	110	110
Potassium Chloride	7.3	7.3
Sodium hydrogen carbonate	29.5	29.5
Sodium Chloride	120	120
SDS	15	15
Sodium nitrate	4.0	4.0
Sodium Sulfate	100	100
Yeast extract	49.0	49.0

**Table 2:** Sampling aging duration.

Testing Period	Test Duration
1	After: 4 Weeks
2	8 Weeks
3	16 Weeks
4	32 Weeks
5	52 Weeks

**Table 3:** Nanotube parameters.

Material	Parameters	Layer Thickness (µm)	Tube Diameter (nm)	Wall Thickness (nm)	Aspect Ratio
TiO_2_NT-O	70 V, 1 h	9.99 ± 0.48	85–125	10.0 ± 2.00	80–117
TiO_2_NT-S	20 V, 6 h	1.45 ± 0.07	60–100	14 ± 2.00	14–24

## Data Availability

Not applicable.
